# A Novel Interference Suppression Method for Interrupted Sampling Repeater Jamming Based on Singular Spectrum Entropy Function

**DOI:** 10.3390/s19010136

**Published:** 2019-01-02

**Authors:** Muyao Yu, Shengbo Dong, Xiangyu Duan, Shangchao Liu

**Affiliations:** 1 Beijing Institute of Remote Sensing Equipment, Beijing 100854, China; shbdong@aliyun.com (S.D.); 13651394075@139.com (S.L.); 2 School of Information and Electronics, Beijing Institute of Technology, Beijing 100081, China; 2220160325@bit.edu.cn

**Keywords:** interrupted sampling repeater jamming (ISRJ), interference suppression, adaptive multi-scale segmentation (AMS), singular spectrum entropy function (SSEF), band-pass filtering

## Abstract

As a new type of jamming, the interrupted sampling repeater jamming (ISRJ) derived from the digital radio frequency memory (DRFM) technology, can generate coherent multiple false targets after pulse compression. At present, the traditional interference suppression method and its improved methods have insufficient characteristics and poor detection performance under the condition of low signal-to-noise ratio (SNR). Aiming at addressing this defect, this paper proposes an interference suppression method for ISRJ based on singular spectrum entropy function (SSEF) from the aspects of singular value decomposition (SVD) and information entropy theories. In this method, firstly, considering the local fine characteristics and extraction efficiency, an adaptive multi-scale segmentation (AMS) method is proposed. The purpose of this processing is to extend the salient characteristics while to smooth the similar ones. In AMS, the segmentation criterion based on average energy of segments and the constraint of minimum segmentation is also proposed, then the improved delay embedded matrix is established from the improved trajectory matrix by AMS and delay embedded mapping. Secondly, the singular spectrum of the improved delay embedded matrix is extracted by SVD. Thirdly, because the recognition algorithms based on singular spectrum analysis (SSA), classical SSE and other characteristics fail at low SNR, this paper proposes a characteristic named as SSEF retrieved from the Shannon entropy model. The following proposed entropy-based threshold detection is carried out on the echo signal to realize the band-pass filtering and interference suppression. Finally, experiment results show that in comparison with other interference suppression approaches, SSEF can increase the probability of target detection and the peak-to-side-lobe ratio (PSR) after pulse compression, which validates its stability to noise and jamming especially in the condition of low SNRs.

## 1. Introduction

Electronic countermeasure (ECM) techniques such as active deception jamming aim at forming false targets to confuse the victim radar, for surrounding desired radar echoes with so many false targets that the target information cannot be extracted. ECM techniques are often enhanced by using digital radio frequency memory (DRFM) systems. In a DRFM system, the interrupted radar signal is down converted first and sampled with a high-speed analogue-to-digital converter (ADC). The samples stored in memory are manipulated in amplitude, frequency and phase to generate the deception jamming, and subsequently processed by a digital-to-analogue converter (DAC), up converted and transmitted back to the victim radar.

As a classic jamming derived from DRFM, the interrupted sampling repeater jamming (ISRJ) is highly correlated to the radar signal which makes it prone to generate multiple false targets after pulse compression [1]. Meanwhile, ISRJ has become more and more ubiquitous with the developed sampling, storage and repeater hardware in DRFM and therefore a great number of the improved interference algorithms are proposed based on related researches, which includes interrupted sampling and direct repeater jamming (ISDRJ), interrupted sampling and periodic repeater jamming (ISPRJ), interrupted sampling and cyclic repeater jamming (ISCRJ) [2,3,4,5], and new jamming patterns based on ISRJ such as frequency shift modulation [6], smart noise modulation [7], and interrupted sampling signal modulation [8,9,10]. ISDRJ, ISPRJ and ISCRJ are distinguished from each other by different repeater strategies which can generate different kinds of false targets after pulse compression. The frequency-shifting deceptive jamming to LFM pulse compression radar generates the forward and backward shifting false targets by the frequency shifting on the view of group delay. The intercepted slices are frequency shift modulation before being transmitted, thus the jamming can achieve better effects for LFM signals by increasing the number of false targets and disordering their positions. Smart noise is also a famous jamming pattern based on ISRJ. When a jammer receives a radar transmitting signal, it performs the convolution with the video noise signal which combines false target pulse train with random noise, then retransmits the jamming signal to victim radar. Interrupted sampling signal modulation controls the false targets information by delay, frequency and phase modulation to achieve pull-off jamming. The active deception jamming based on ISRJ is highly coherent to the radar transmitting signal, which makes the detection of the deception jamming difficult.

In comparison with jamming technology, the anti-jamming technique turns out to be a more arduous task due to the shortage of prior signal information and published researches, as well as military sensitivity and no related theoretical frameworks and mathematical models [11]. Despite this, in depth studies are still carried out on interference suppression methods. According to [12], based on the generalized periodicity of the pseudo-random sequence modulated radar signal, its characteristics can be extracted by the eigenvector analysis which was further used for the independent component analysis (ICA). This method successfully separated the radar echo signal from the jamming signal and the recognition probability of which can be improved by multiple ICA operations, but it only applies to the condition of non-coherent interference signal and large number of ICA operations need to be performed if the jamming-to-signal ratio (JSR) is high. The Sliding-Truncation Matched Filter (STMF) method proposed in [13] first extracted the two-dimensional amplitude distribution after pulse compression by searching for the reference window width and delay of the matched filter. Based on that, the jamming slice width and the repeater period were estimated for further interference suppression. The advantage of this method is that it can accurately identify and suppress different ISRJ strategies in condition of high JSR. However, for low JSR, the jamming recognition capability of this method declines rapidly and the time efficiency becomes lower because the estimation is carried out after the two-dimensional searching. Utilizing the discontinuity of the ISRJ in the frequency domain, the improved band-pass filtering interference suppression methods based on the energy function through the short time Fourier transform (STFT) were proposed in [14,15]. These methods reduce the effects from the noise signal and improve interference suppression performance in condition of low SNR. However, considering that the minimum value of the energy function needs to be segmentally searched in time domain or time-frequency domain, therefore, their calculation volume is rather large. In [16], an interference suppression method based on the improved matching tracking and base tracking was proposed by the multi-parameter atomic decomposition of the jamming signal and the subtle feature extraction including the expansion coefficient and the primary atomic parameter set. This method can achieve high jamming recognition probability with low SNR, but the selection of a suitable complete atomic library and the extraction of the effective parameters become a problem which makes it difficult to apply in practical engineering. In [17], an energy function detection and band-pass filtering (EFDBP) method was proposed, which constructed an energy function and extracted the undisturbed signal segments by setting detection thresholds. These signal segments were used to construct a band-pass filter in the frequency domain which was windowed to optimize the matching filtering effect. The method is simple in structure and convenient in engineering application, but the interference suppression effect is unstable not only in the condition of low SNR but low JSR, and meanwhile the performance of this method can be significantly affected by different filter windows. Based on EFDBP, the compressed sensing theory was introduced into the interference suppression which regarded the band-pass filtered signal as the compressed data and constructed a minimum solution model using the linear relationship between compressed data and the echo signal in sparse frequency domain after the de-linearization process [18]. Moreover, the orthogonal matched filter tracking algorithm was further applied to reconstruct the echo signal and finally realized the interference suppression. This method successfully suppresses the interference to some extent and reconstructs the echo signal without distortion. However, the performance of this method changes with different ISRJ strategies and is particularly sensitive to the width of the time sampling window. Experiment results show that this method is completely unable to suppress the interference with small time sampling window and there will be significant phase distortion in the reconstructed echo signal. In [19,20], a particle filter (PF) anti-jamming method was proposed, which updated the band-pass filter in real time by detecting the abnormal changes of the importance weights during the particle filter process. This method is relatively practical because it doesn’t need to estimate the state transfer function and the measurement noise of the system, and meanwhile less sensitive to the JSR which solves the low target detection probability problem. However, this method is more likely to be affected by system which limits its application in real scenario. In [21,22], the waveform parameters were adjusted in real time by adaptive intra-pulse orthogonal coding and waveform diversity, so that the jamming signal and the target signal were orthogonal at the radar receiver and based on that the interference suppression could be further performed by the matched filter. This method tried to realize the interference suppression from the aspect of the transmitting system which makes it possible to separate the jamming signal and the target signal completely. However, in this method, the transmit power is reduced because of the interposition of the screen signal and meanwhile the performance of interference suppression will also degrade because it is usually applicable to the condition that jamming lags one or more pulse repetition periods.

In summary, most interference suppression methods are based on the idea of characteristics extraction, difference identification and band-pass filter construction. These methods perform well under the high SNR and high JSR condition, but when comes to low SNR, their performances will degrade. Aiming at this defect, this paper proposed an interference suppression method based on SSEF for ISRJ from the aspect of entropy characteristics. Firstly, the improved trajectory matrix is established by adaptive multi-scale segmentation (AMS) to achieve fine characteristics extraction of the echo signal and improves time efficiency. In this step, the segmentation criterion based on average energy of segments and the constraint of minimum segmentation is also propose. Secondly, the improved trajectory matrix is delay embedded mapped to develop the improved delay embedded matrix for further SVD. Thirdly, the singular spectrum of the improved delay embedded matrix is extracted. Based on the SSEF retrieved from the Shannon entropy model, threshold detection is carried out on the echo signal to realize the band-pass filtering and the interference suppression. Finally, experiment results show that in comparison with other interference suppression approaches such as EFDBP, STFT, PF, STMF, the proposed method in this paper can increase the probability of target detection and the peak-to-side-lobe ratio (PSR) after pulse compression, which validates its stability to noise and jamming especially in the condition of low SNRs.

The structure of this paper is as follows: in Section 2, the working mode of jammer and the mechanism of ISRJ is introduced, then the echo signal model is established. In Section 3, the interference suppression method based on SSEF is proposed, and the key steps of data segmentation and characteristics extraction are described in detail. In Section 4, simulation comparison experiments are carried out to quantitatively evaluate the interference suppression performances. Finally, the main conclusion and directions for future research are presented in Section 5.

## 2. The Mechanism of ISRJ

### 2.1. The Working Mode of Jammer

**Figure 1:**
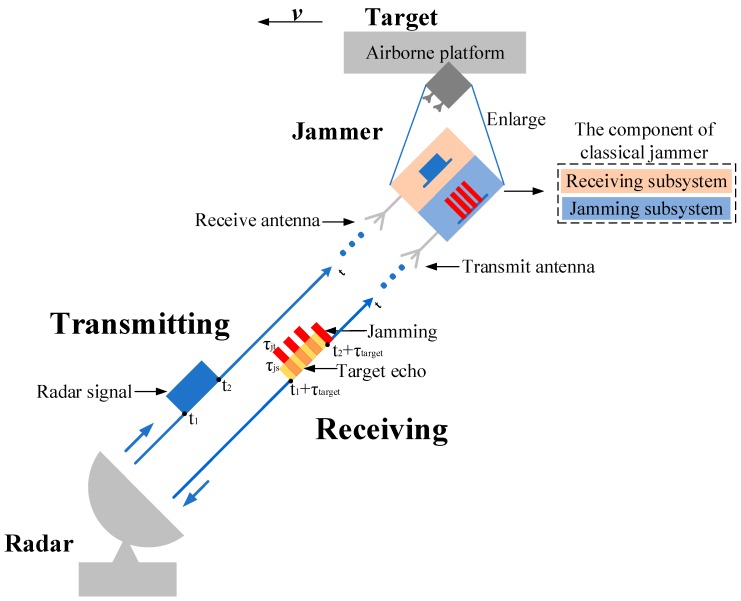
The schematic diagram of jammer against radar based on ISRJ.

In order to introduce jamming more clearly, we take the airborne jammer on the condition of self-screening jamming (SSJ) as an example. In general, the jammer is divided into receiving subsystem and jamming subsystem. The receiving subsystem includes a receive antenna, a receiver and a signal processer while the jamming subsystem includes a transmit antenna, a jamming generator and a power control unit. The jammer receives the radar transmitting signal through the receive antenna after detecting the rising edge of the radar transmitting signal and generates slices by interrupted sampling, storing. Then, after modulation, the ISRJ is transmitted through the transmit antenna to victim radar. By this way, the ISRJ based on DRFM generates main false target and several other false targets located symmetrically around the main false target. The schematic diagram of jammer against radar based on ISRJ is showed in Figure 1. The rectangle in blue corresponds to the radar transmitting signal, the rectangle in yellow corresponds to the target signal and the rectangles in red correspond to ISRJ. The radar transmits a signal which pulse width is ($t_{2}-t_{1}$). After detection and jamming generation, the echo signal includes target signal, jamming signal and noise. Here, the noise is ignored in the diagram for convenience. The delay of the target signal is $\tau_{\mathrm{target}}$ because the target distance is $\frac{1}{2}{c\tau }_{\mathrm{target}}$ where *c* is velocity of light. The pulse width of the interrupted sampling is $\tau_{\mathrm{js}}$ and the pulse width of the repeater is $\tau_{\mathrm{jt}}$. The amplitude of the target signal is smaller than the transmitting signal due to the distance and the amplitude of the jamming is greater than target signal due to JSR. Further, the overlap of the two signals causes the amplitude to become larger.

### 2.2. The Principle of ISRJ

The ISRJ derived from signal sampling, storage and repeater can generate coherent multiple false targets during the matched filtering of pulse compression. At present, the papers and researches in the field of interference suppression mainly focus on pulse Doppler (PD) radar and linear frequency modulation (LFM) radar. During the process of interference suppression, signal accumulation is needed. The PD signal performs pulse accumulation in the time dimension while LFM signal performs pulse compression, which is equivalent to accumulation. Considering that the bandwidth of LFM signal is relatively wide and the resolution is high, LFM signal has great advantages for target detection [23]. Both waveforms are used in engineering applications while the interference suppression method in this paper can extract more characteristics by using LFM signal, so the main research in this paper is to deal with the interference suppression under LFM signal. Here, we assume that the transmitting radar signal is LFM signal with expression as:

(1) $$s(t)=u(t)e^{j2\pi (f_{0}+\frac{1}{2}Kt)t}=Arect(\frac{t}{T})e^{j2\pi (f_{0}+\frac{1}{2}Kt)t}$$

where *A* is the amplitude, *T* is the pulse width, $f_{0}$ is the carrier frequency, and *K* is the frequency modulation slope.

The interrupted sampling signal is rectangular pulse train which can be written as:

(2) $$p(t)=rect(\frac{t}{\tau })\cdot \sum_{n=-\infty }^{+\infty }\delta (t-nT_{s})=\sum_{n=-\infty }^{+\infty }rect(\frac{t-nT_{s}}{\tau })$$

where τ, $T_{s}$ and $f_{s}=\frac{1}{T_{s}}$ respectively stand for the pulse width, the period and the frequency of the sampling signal in jammer.

After interrupted sampling, the jamming signal can be expressed as:

(3) $$j(t)=s(t)\cdot p(t)=s(t)\cdot \sum_{n=-\infty }^{+\infty }rect(\frac{t-nT_{s}}{\tau })=\frac{\tau }{T_{s}}\cdot s(t)+\frac{2\tau }{T_{s}}\sum_{n=-\infty }^{+\infty }\frac{\sin(\pi nf_{s}\tau )}{\pi nf_{s}\tau }\cdot \cos(2\pi nf_{s}t)\cdot s(t)$$

After DAC, up converted and transmitted back to the victim radar, the echo signal received by the radar receiver can be indicated as:

(4) $$r(t^{\prime })=s(t^{\prime })+j(t^{\prime })+n(t^{\prime })$$

where *s*(*t*′) represents the echo signal, *j*(*t*′) is the jamming signal and *n*(*t*′) is the Gaussian noise. The time sequence of the radar receiver has a delay relative to that of the jammer.

**Figure 2:**
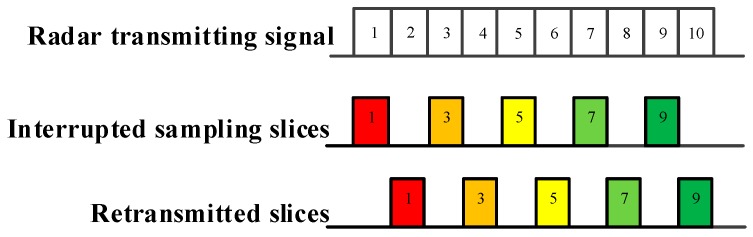
Mechanism of interrupted sampling and direct repeater jamming (ISDRJ).

**Figure 3:**
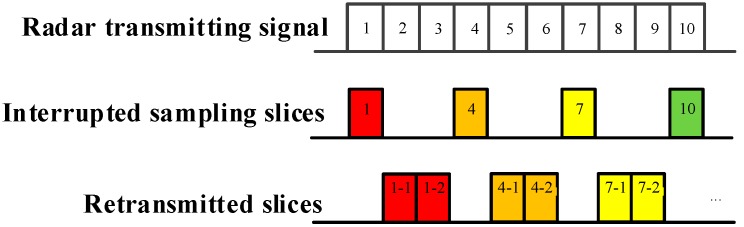
Mechanism of interrupted sampling and periodic repeater jamming (ISPRJ).

**Figure 4:**
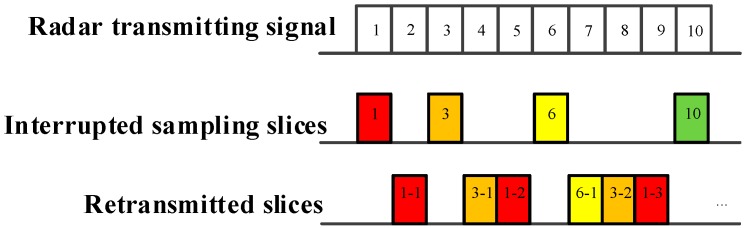
Mechanism of interrupted sampling and cyclic repeater jamming (ISCRJ).

As demonstrated in Figure 2, Figure 3 and Figure 4, ISRJ can be divided as ISDRJ, ISPRJ and ISCRJ by different sampling and repeater strategies. Different colored rectangles correspond to different jamming slices. Jamming signals are first sampled by different interrupted sampling signals to generate different patterns. If the repeater time window is the same as the sampling time window with 0.5 duty ratio, then ISDRJ is produced by the sampling and repeater loop right after intercepting the slice of the radar signal. Unlike ISDRJ, ISPRJ periodically repeats a certain number of the jamming slices (set by program) after sampling, then the sampling and repeater loop is applied until the signal ends. Besides that, the ISCRJ repeats the current jamming slices and the previous jamming slices in order according to the repeating period until the end of the signal which may leads to numerous multiple false targets after matched filtering.

The above Equation (3) is converted into matrix for further derivation. Here, we assume that the radar transmitting signal vector is $s={\left[s_{1}\;s_{2}\;\ldots \;s_{n}\right]}^{T}$, where $s_{i}$, *i* = 1, 2, …, *n* are the sample values of the radar signal with $f_{s}$ in jammer. Based on that, the interrupted sampling signal can be derived as:

(5) $$j_{1}=Ps$$

where ***P*** is the interrupted sampling matrix. ***P*** is a diagonal matrix and the values of the diagonal elements are ‘one’ or ‘zero’ [22]. The element ‘one’ means the intercepting processing, while the element ‘zeros’ means the retransmitting processing.

It should be noted that the expressions of the interrupted sampling matrix are different with strategies. In this paper, the interrupted sampling matrix of ISDRJ, ISPRJ and ISCRJ are respectively named as $P_{\mathrm{ISDRJ}}$, $P_{\mathrm{ISPRJ}}$ and $P_{\mathrm{ISCRJ}}$ in Equations (6), (7) and (11):

(6) $$P_{\mathrm{ISDRJ}}=\left[\begin{array}{ccccc} I_{{tf}_{s}\times {tf}_{s}} & & & & \\ & 0_{tf_{s}\times {tf}_{s}} & & & \\ & & I_{{tf}_{s}\times {tf}_{s}} & & \\ & & & 0_{tf_{s}\times {tf}_{s}} & \\ & & & & \ddots \end{array}\right]$$

(7) $$P_{\mathrm{ISPRJ}}=\sum_{i=1}^{N}P_{i}$$

where ***I*** is the unit matrix, **0** is zero matrix, τ and $f_{s}$ are the pulse width and the pulse repetition frequency (PRF) of the sampling signal in jammer, *N* is the time of repeater. With the initial interrupted sampling matrix $P_{0}$ composed by sub-matrix including unit matrixes and zero matrixes from Equation (8) and the transition matrix ***B*** from Equation (9), $P_{i}$ can be further deduced as Equation (10):

(8) $$P_{0}=\left[\begin{array}{ccccc} I_{{tf}_{s}\times {tf}_{s}} & & & \\ & 0_{{Ntf}_{s}\times {Ntf}_{s}} & & & \\ & & I_{{tf}_{s}\times {tf}_{s}} & & \\ & & & 0_{{Ntf}_{s}\times {Ntf}_{s}}\\ & & & & \ddots \end{array}\right]$$

(9) $$B=\left[\begin{array}{cc} I_{n\times n} & ,0_{n\times ({i\tau f}_{s}+n)} \end{array}\right]$$

(10) $$P_{i}=B\left[\begin{array}{cccc} 0_{{\tau f}_{s}\times {\tau f}_{s}} & & & \\ & \overset{\left(i\right)}{\ddots } & & \\ & & 0_{{\tau f}_{s}\times {\tau f}_{s}} & \\ & & & P_{0} \end{array}\right]B^{T}$$

(11) $$P_{\mathrm{ISCRJ}}=\sum_{i=1}^{N}{{CP}_{i}}^{\prime }$$

Assuming that $P_{0}^{\prime }$ is the initial interrupted sampling matrix of ISCRJ composed by unit matrixes and zero matrixes and ***C*** is the loop matrix, then $P_{i}^{\prime }$ can be written as Equation (13):

(12) $$P_{0}^{\prime }=\left[\begin{array}{ccccc} I_{\tau f_{s}\times \tau f_{s}} & & & & \\ & 0_{\tau f_{s}\times \tau f_{s}} & & & \\ & & \ddots & & \\ & & & I_{N\tau f_{s}\times N\tau f_{s}} & \\ & & & & 0_{N\tau f_{s}\times N\tau f_{s}} \end{array}\right]$$

(13) $$P_{i}^{\prime }=B\left[\begin{array}{cccc} 0_{\tau f_{s}\times \tau f_{s}} & & & \\ & \overset{\left(i\right)}{\ddots } & & \\ & & 0_{\tau f_{s}\times \tau f_{s}} & \\ & & & P_{0}^{\prime } \end{array}\right]B^{T}$$

The spectrum $J_{1}$ is derived from the Fourier transform of the initial interrupted sampling signal $j_{1}$:

(14) $$J_{1}=F_{1}j_{1}=\left[\begin{array}{cccc} e^{-j2\pi f_{1}t_{1}} & e^{-j2\pi f_{1}t_{2}} & \cdots & e^{-j2\pi f_{1}t_{n}}\\ e^{-j2\pi f_{2}t_{1}} & e^{-j2\pi f_{2}t_{2}} & \cdots & e^{-j2\pi f_{2}t_{n}}\\ \vdots & \vdots & \ddots & \vdots \\ e^{-j2\pi f_{n}t_{1}} & e^{-j2\pi f_{n}t_{2}} & \cdots & e^{-j2\pi f_{n}t_{n}} \end{array}\right]j_{1}$$

where $F_{1}$ is the Fourier transform matrix. The spectrum of the jamming signal can be generated by the time delay $\tau_{0}$:

(15) $$J_{2}=\Gamma J_{1}=\left[\begin{array}{cccc} e^{-j2\pi f_{1}\tau_{0}} & 0 & \cdots & 0\\ 0 & e^{-j2\pi f_{2}\tau_{0}} & \cdots & 0\\ \vdots & \vdots & \ddots & \vdots \\ 0 & 0 & \cdots & e^{-j2\pi f_{n}\tau_{0}} \end{array}\right]J_{1}$$

After the inverse Fourier transform, the jamming signal ***j*** can be derived as Equation (16):

(16) $$j=F_{2}J_{2}=\left[\begin{array}{cccc} e^{j2\pi f_{1}t_{1}} & e^{j2\pi f_{2}t_{1}} & \cdots & e^{j2\pi f_{n}t_{1}}\\ e^{j2\pi f_{1}t_{2}} & e^{j2\pi f_{2}t_{2}} & \cdots & e^{j2\pi f_{n}t_{2}}\\ \vdots & \vdots & \ddots & \vdots \\ e^{j2\pi f_{1}t_{n}} & e^{j2\pi f_{2}t_{n}} & \cdots & e^{j2\pi f_{n}t_{n}} \end{array}\right]J_{2}$$

where $F_{2}$ is the inverse Fourier transform matrix.

Therefore, the ISRJ signal matrix can be summarized as Equation (17). In order to facilitate the theoretical derivation and simulation, we set radar sampling frequency $f_{sr}=f_{s}$, so radar echo signal matrix can be summarized as Equation (18):

(17) $$j=F_{2}\Gamma F_{1}Ps$$

(18) $$r=s+j+n=s+F_{2}\Gamma F_{1}\mathrm{Ps}+n=(I+F_{2}\Gamma F_{1}P)s+n=As+n$$

where ${A=I+F}_{2}{\Gamma F}_{1}P$. and the radar receiver can’t be saturated by the jamming signal.

## 3. SSEF for ISRJ Suppression

Based on the above echo signal model, this paper proposes an SSEF-based ISRJ interference suppression method which first generates an improved delay embedded matrix through the AMS and the delay embedded mapping. After that, a singular spectrum model is applied to realize the SVD of this matrix. Related SSEF can be extracted by the establishment of the Shannon entropy model along with the threshold detection to realize the band-pass filtering of the jamming in the echo signal.

### 3.1. AMS

The performance of the SSEF-based interference suppression method mainly relies on the width of the segmentation window during the AMS and the reconstruction principle of the delay embedded mapping space.

#### 3.1.1. Fixed-Scale Segmentation Window

In this section, the effect of the width about segmentation window is first discussed. Assuming that the signal sequence detected at the radar receiver is $r={\left[r_{1}\;r_{2}\;\ldots \;r_{n}\right]}^{T}$ using radar sampling frequency $f_{sr}$ with the width of segmentation window set as an integer l∈[1,n]. The trajectory matrix ***R*** of signal sequence ***r*** is formed [24]. Therefore, ***R*** can be deduced as:

(19) $$R=\left[\begin{array}{cccc} r_{1} & r_{2} & \cdots & r_{n-l+1}\\ r_{2} & r_{3} & \cdots & r_{n-l+2}\\ \vdots & \vdots & \ddots & \vdots \\ r_{l} & r_{l+1} & \cdots & r_{n} \end{array}\right]=\left[\begin{array}{cccc} R_{1} & R_{2} & \cdots & R_{n-l+1} \end{array}\right]$$

where $R_{i}={\left[r_{i}\;r_{i+1}\;\ldots \;r_{i+l-1}\right]}^{T}$, *i* = 1, 2, …, *n* − *l* + 1.

Considering that the SSEF needs to be calculated based on the singular value after the segmentation reconstruction, there won’t be any local optimum error with long segmentation window because the large data volume can smooth the SSEF curve and make it more suitable for threshold detection. However, in this condition, the calculated SSE is of low time efficiency. Similarly, near real-time calculation can be achieved with short segmentation window because of the small data volume and meanwhile the SSE curve contains more details. But the local optimum error can lead to burrs in the signal edges after interference suppression processing. In summary, the width of the segmentation window can be written as:

(20) $$l=\alpha Tf_{sr}$$

where *T* is the pulse width of the radar transmitting signal, $f_{sr}$ is the PRF of radar and α is the adjusted factor determined by the slice width of the jamming with common value ranges from 0.02 to 0.25.

#### 3.1.2. Adaptive Multi-Scale Segmentation Window

Considering that the target and the jamming in the radar echo signal are sparse relative to the noise and the recognition probability of the noise is high, short segmentation window is not required for noise. However, the short segmentation window is more advantageous for extracting the fine characteristics when it comes to the target signal or the jamming signal. It is necessary to design a reasonable window for local characteristics extraction. At the same time, the fixed width segmentation window will also lead to the decline of time efficiency. In order to solve this problem, this paper proposes an AMS method, which adaptively changes the width of the segmentation window to achieve fine characteristics extraction of the echo signal and improves time efficiency.

Assuming that $l_{1}{=\alpha Tf}_{sr}$ is the initial width of segmentation window, repeat the following AMS criterion algorithm and update the variable *i* until all segmentation is completed. This process is constrained by $l_{i}\geq \frac{l_{1}}{2}$:

(21) $$\{\begin{array}{cc} l_{i}=l_{i}+1 & E(R_{i}^{\prime })\leq E_{L}\\ \begin{array}{l} l_{i}=l_{i}-1\\ l_{i}=l_{i} \end{array} & \begin{array}{l} E(R_{i}^{\prime })\geq E_{U}\\ others \end{array} \end{array}$$

where $l_{i}$ is the width of *i*-th segmentation window, ${E(R}_{i}^{\prime })$, $E_{L}$, $E_{U}$ respectively stand for the average energy of the *i*-th segment, lower threshold and upper threshold. They can be expressed as:

(22) $$E(R_{i}^{\prime })=\frac{1}{l_{i}}\sum_{p=1}^{l_{i}}{\left|r_{p}\right|}^{2}$$

(23) $$E_{L}=\frac{1}{N}\sum_{q=1}^{N}{\left|r_{q}\right|}^{2}$$

(24) $$E_{U}=\frac{\frac{1}{N}\sum_{t=1}^{N}{\left({\left|r_{t}\right|}^{2}-E_{L}\right)}^{2}}{E_{L}}$$

When ${E(R}_{i}^{\prime })$ is less than $E_{L}$, the signal in the segment does not float much. The signal in this segment is a noise signal and its characteristics are relatively obvious. The segmentation window is adjusted to a long window, which is used to smooth the noise. When ${E(R}_{i}^{\prime })$ is greater than $E_{U}$, it indicates that the floatability of the signal in this segment is very large. The segmentation window is adjusted to a short window and the fine characteristics of the signal are extracted. Meanwhile, the short segmentation window is beneficial to improve the time efficiency. Since the widths of the segments after the AMS are different, the number of matrix rows is kept the same ($l_{\max}$) by the zero-padding operation. This step does not affect SVD and entropy extraction. In summary, the improved trajectory matrix formed by the AMS algorithm can be written as:

(25) $$R^{\prime }=\left[\begin{array}{cccc} r_{1} & r_{2} & \cdots & r_{n-\hat{l}+1}\\ r_{2} & r_{3} & \cdots & r_{n-\hat{l}+2}\\ \vdots & \vdots & \ddots & \vdots \\ \begin{array}{l} r_{l_{1}}\\ 0\\ \vdots \\ 0 \end{array} & \begin{array}{l} r_{l_{2}}\\ 0\\ \vdots \\ 0 \end{array} & \begin{array}{l} \cdots \\ \cdots \\ \ddots \\ \cdots \end{array} & \begin{array}{l} r_{l_{n}}\\ 0\\ \vdots \\ 0 \end{array} \end{array}\right]=\left[\begin{array}{cccc} R_{1}^{\prime } & R_{2}^{\prime } & \cdots & R_{n-\hat{l}+1}^{\prime } \end{array}\right]$$

where $\hat{l}$ is the number of the segment after AMS and ***R***′ is a matrix of $l_{\max}\times (n-\hat{l}+1)$.

### 3.2. Delay Embedded Mapping

After AMS, the improved trajectory matrix ***R***′ is delay embedded mapped in column through nonlinear transformation to develop the delay embedded matrix ***D*** for further SVD [25]. The delay embedded matrix can be written as:

(26) $$D=\left[\begin{array}{cccc} D_{1} & D_{2} & \cdots & D_{n-\hat{l}+1} \end{array}\right]=f(R^{\prime })=f\left(\left[\begin{array}{cccc} R_{1}^{\prime } & R_{2}^{\prime } & \cdots & R^{\prime }{}_{n-\hat{l}+1} \end{array}\right]\right)=\left[\begin{array}{cccc} f(R_{1}^{\prime }) & f(R_{2}^{\prime }) & \cdots & f(R^{\prime }{}_{n-\hat{l}+1}) \end{array}\right]$$

with $D_{i}{=f(R}_{i}^{\prime })$, $i=1,\;2,\;\ldots ,\;n-\hat{l}+1$, where *f*(•) is the delay embedded mapping which can cause different singular value of the delay embedded matrix after reconstruction. The delay embedded mapping can be indicated as:

(27) $$D_{i}=f(R_{i}^{\prime })=\left[\begin{array}{cccc} r_{i} & r_{i+1} & \cdots & r_{w}\\ r_{i+1+d} & r_{i+2+d} & \cdots & r_{w+1+d}\\ \vdots & \vdots & \ddots & \vdots \\ r_{l_{\max}-w+1} & r_{l_{\max}-w+2} & \cdots & r_{i+l_{\max}-1} \end{array}\right]$$

where ${w=l}_{\max}-w+1$ and *d* is the delay time responsible for the component of the delay embedded matrix.

When *d* = 0, $D_{i}$ turns into Hankel matrix with equivalent $a_{i,j}$ at *i + j = c* where *c* is constant [26]. Considering that the singular value of the Hankel matrix is relatively obvious, therefore it can achieve better performance during the noise reduction process. However, the extraction results cover all singular values which will increase the computation burden. In contrary, when *d* > 0, only main singular values are extracted and the number of which is determined by the row number. In this condition, the computation burden is relatively smaller, but it will generate ambiguous singular values and therefore deteriorate the performance of noise reduction.

As to ensure that all elements participate in the operation, *d* should belong to [0, *w*]. In this paper, *d* is set as 2 to solve the compromise problem between the calculation burden and the singular value extraction. Then the improved delay embedded mapping can be expressed as:

(28) $$D_{i}=f(R_{i}^{\prime })=\left[\begin{array}{cccc} r_{i} & r_{i+1} & \cdots & r_{w}\\ r_{i+3} & r_{i+4} & \cdots & r_{w+3}\\ \vdots & \vdots & \ddots & \vdots \\ r_{l_{\max}-w+1} & r_{l_{\max}-w+2} & \cdots & r_{i+l_{\max}-1} \end{array}\right]$$

### 3.3. SVD

SVD is an important tool in matrix analysis which reflects the intrinsic nature of matrix vectors in statistics [27]. The main application of SVD is principal component analysis (PCA) by mapping data to the lower dimensional space and extracting data features in order of importance. Larger singular value indicates more obvious characteristics which can be further used to the sortation and the identification of the signal. Therefore, SVD is equipped with good numerical stability and geometric invariance.

According to the SVD theory [28], for each improved delay embedded matrix $D_{i}$, there will be an orthogonal matrix ***U***, ***V*** to realize:

(29) $$D_{i}=U\Sigma_{i}V^{T}$$

Thus, the singular spectrum of each improved delay embedded matrix is:

(30) $$\Sigma_{i}=\mathrm{diag}\left(\begin{array}{cccc} \sigma_{i,1} & \sigma_{i,2} & \cdots & \sigma_{i,l_{\max}-w+1} \end{array}\right)$$

The singular values in singular spectrum represent the relevant characteristics contained in segments of radar echo signal. In fact, after a certain singular value, the latter values are very small, which means that the important characteristics are former ones and the rest are secondary characteristics.

### 3.4. Shannon Entropy Model

Information entropy is widely applied to measure the complexity of the signal which is proportional to the signal uncertainty [29]. Therefore, the information of singular spectrum can be estimated by calculating the SSE. Considering that the Gaussian distribution is equipped with the largest information quantity among different random variables, thus it has the maximum information entropy. Since different targets have different radar cross sections (RCS), the amplitude of the radar echo signal is also subject to a certain random distribution. Moreover, the amplitude of the jamming signal derived from the interrupted sampling is usually a large fixed value because of the JSR, and the information contained in the jamming signal is much smaller than the Gaussian noise and the echo signal which means its information entropy is significantly different from the other two. This can be further applied to the jamming signal extraction and the noise reduction. In this paper, the Shannon entropy is assumed to be the entropy characteristics of the singular spectrum, therefore the singular spectrum entropy of *i*-th delay embedded matrix is:

(31) $$H_{\mathrm{SVD}}(\Sigma_{i})=-\sum_{j=1}^{l_{\max}-w+1}p_{i,j}\log_{2}p_{i,j}$$

where $p_{i,j}$ is the probability of the *j*-th singular value in the singular spectrum of the *i*-th improved delay embedded matrix. This probability represents the proportion of each singular value in singular spectrum. For the noise, since the relative variation of singular values in singular spectrum is small, the probability of each singular value is not much different. According to the principle of entropy, SSE is a large value. For the target signal, the difference between singular values begin to increase due to RCS, which make SSE decrease. But due to the influence of target location and attenuation in transmitting, SSE does not decrease much. And for ISRJ, the amplitude of ISRJ is usually much greater than target signal and noise, so in this case there are only a few large singular values in the entire singular spectrum, which causes the difference between the probability of these large singular values and that of the rest to be much greater. Therefore, the SSE becomes much smaller than that of noise and target.

(32) $$p_{i,j}=\frac{\sigma_{i,j}}{\sum_{j=1}^{l_{\max}-w+1}\sigma_{i,j}}$$

According to Equations (31) and (32), as to ensures that SSEF make sense, *p_i,j_* should be non-zero. The proof is shown in Appendix A for details. Then, the entropy-based threshold is set to extract the undisturbed target signal. The threshold detection algorithm is demonstrated as Equation (33) where $H_{\mathrm{SVD}}{(D}_{i})$ is the SSE of the *i*-th improved delay embedded matrix:

(33) $$\{\begin{array}{cc} r_{l_{i}}=0 & ,\;H_{\mathrm{SVD}}(\Sigma_{i})<\gamma \\ {\hat{r}}_{l_{i}}=r_{l_{i}} & ,H_{\mathrm{SVD}}(\Sigma_{i})\geq \gamma \end{array}$$

where ${\hat{r}}_{l_{i}}$ is the element after interference suppression, $r_{l_{i}}$ is the last non-zero element of the column in the improved trajectory matrix. The threshold can be expressed as:

(34) $$\gamma =\beta \frac{1}{N}\sum_{i=1}^{N}H_{\mathrm{SVD}}(\Sigma_{i})$$

where β is the threshold adjusted factor. Generally, the first *N* delay embedded matrix only contains the noise without any components of the target signal and the jamming signal. Empirically, β ranges from 0.8 to 0.9 and *N* values from 2 to 10. Here, β = 0.88, *N* = 5.

Finally, the signal after interference suppression $r_{a}$ can be retrieved from the updated improved trajectory matrix and the recovered signal sequence by Equation (33).

### 3.5. Algorithm Procedure

**Figure 5:**
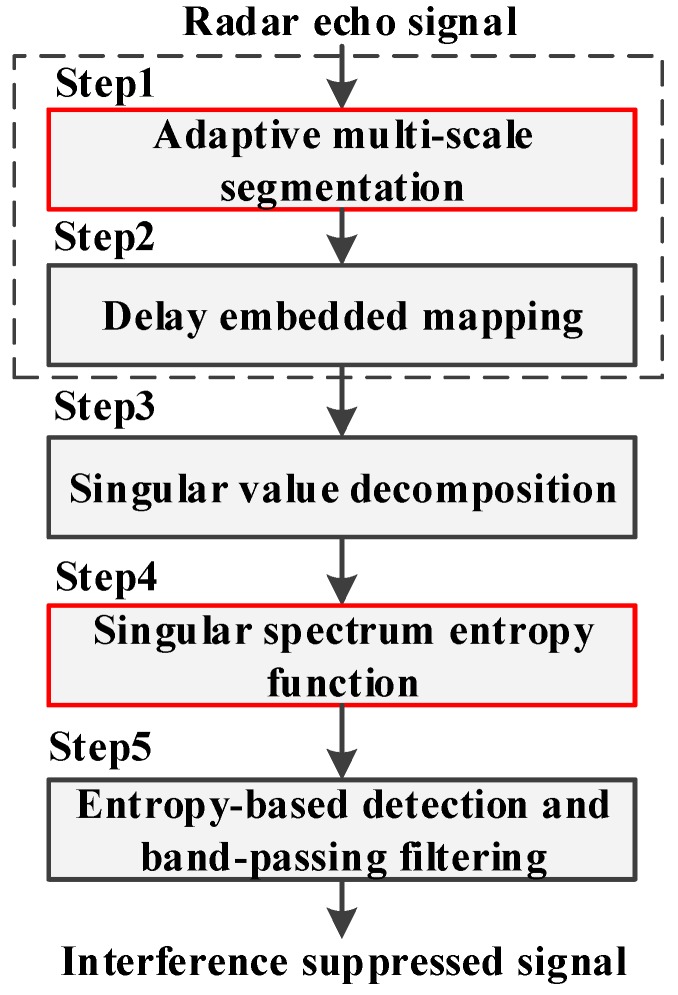
Flow chart of interference suppression based on SSEF.

The flow chart of the SSEF-based ISRJ interference suppression method can be summarized as Figure 5. The specific steps can be expressed as follows:

Step 1: Computing the improved trajectory matrix ***R***′. The echo signal sequence ${r=[r}_{1}{\;r}_{2}{\;\ldots \;r}_{n}{]}^{T}$ received at the radar antenna is processed by AMS to generate the improved trajectory matrix ***R***′. The mathematical expression is rewritten here:

(35) $$R^{\prime }=\left[\begin{array}{cccc} r_{1} & r_{2} & \cdots & r_{n-\hat{l}+1}\\ r_{2} & r_{3} & \cdots & r_{n-\hat{l}+2}\\ \vdots & \vdots & \ddots & \vdots \\ \begin{array}{l} r_{l_{1}}\\ 0\\ \vdots \\ 0 \end{array} & \begin{array}{l} r_{l_{2}}\\ 0\\ \vdots \\ 0 \end{array} & \begin{array}{l} \cdots \\ \cdots \\ \ddots \\ \cdots \end{array} & \begin{array}{l} r_{l_{n}}\\ 0\\ \vdots \\ 0 \end{array} \end{array}\right]=\left[\begin{array}{cccc} R_{1}^{\prime } & R_{2}^{\prime } & \cdots & R_{n-\hat{l}+1}^{\prime } \end{array}\right]$$

Step 2: Computing the improve delay embedded matrix ***D****. **D*** is written as:

(36) $$D=\left[\begin{array}{cccc} D_{1} & D_{2} & \cdots & D_{n-\hat{l}+1} \end{array}\right]\;=f(R^{\prime })=\left[\begin{array}{cccc} f({R^{\prime }}_{1}) & f({R^{\prime }}_{2}) & \cdots & f({R^{\prime }}_{n-\hat{l}+1}) \end{array}\right]$$

where *f*(•) is the delay embedded mapping defined in Equation (28).

Step 3: Computing the singular spectrum Σ. ${\Sigma =[\Sigma }_{1}{\;\Sigma }_{2}{\;\ldots \;\Sigma }_{n-\hat{l}+1}]$, $\Sigma_{i}$ is computed by SVD in Equation (29) and expressed as:

(37) $$\Sigma_{i}=\mathrm{diag}\left(\begin{array}{cccc} \sigma_{i,1} & \sigma_{i,2} & \cdots & \sigma_{i,l_{\max}-w+1} \end{array}\right)$$

Step 4: Computing the SSEF $H_{\mathrm{SVD}}\left(\Sigma \right)$ by Equation (38). $H_{\mathrm{SVD}}{(\Sigma }_{i})$ is written as Equation (31):

(38) $$H_{\mathrm{SVD}}(\Sigma )=\left[\begin{array}{cccc} H_{\mathrm{SVD}}(\Sigma_{1}) & H_{\mathrm{SVD}}(\Sigma_{2}) & \cdots & H_{\mathrm{SVD}}(\Sigma_{n-\hat{l}+1}) \end{array}\right]$$

Step 5: Updating the element ${\hat{r}}_{l_{i}}$ in improved trajectory matrix ***R***′ by entropy-based detection and band-pass filtering. The specific threshold detection algorithm is described below and entropy-based threshold *γ* is defined in Equation (34). After this step, the interference suppressed signal $r_{a}$ can be retrieved:

(39) $$\{\begin{array}{cc} r_{l_{i}}=0 & ,H_{\mathrm{SVD}}(\Sigma_{i})<\gamma \\ {\hat{r}}_{l_{i}}=r_{l_{i}} & ,H_{\mathrm{SVD}}(\Sigma_{i})\geq \gamma \end{array}$$

## 4. Simulation

### 4.1. Comparison between Fixed-Scale Segmentation and AMS on Characteristcs Extraction

In this experiment, the transmitting radar signal is assumed to be LFM signal as shown in Equation (1). Based on the jamming model in Section 2, related parameters are set as Table 1. Here, we assume that the target is located at 4 km and the target RCS distribution satisfies Swerling IV to simulate fast moving targets such as ships and missiles.

**Figure 6:**
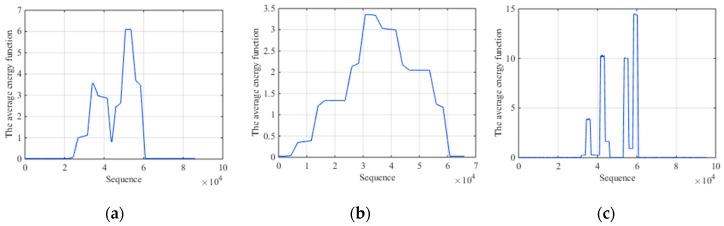
Comparison results with ISCRJ and SNR = 10 dB. (**a**) The average energy function of short fixed-scale segmentation. (**b**) The average energy function of long fixed-scale segmentation. (**c**) The average energy function of AMS.

We first carry out the simulation comparison experiment by extracting the average energy of segments with fixed-scale segmentation and AMS. In order to highlight the comparison results, the experiment is established under the condition of high SNR. That is because under this condition, both two methods are efficient and the advantage of AMS in fine characteristic can be highlighted successfully. Figure 6a is the average energy of each segment by short fixed-scale segmentation with ISCRJ and SNR = 10 dB. Figure 6b is that of long fixed-scale segmentation with the same condition. Figure 6c is the AMS result. It can be seen that the average energy function with AMS has obvious fluctuation while the function with long fixed-scale is smoother. Although the result of short fixed-scale is similar to AMS, it produces burrs in the segments where it should be smooth, which affects the characteristics recognition. Meanwhile, a large number of segmentations will reduce the time efficiency. So AMS has an advantage in fine characteristics extraction and time efficiency relative to fixed-scale segmentation.

**Figure 7:**
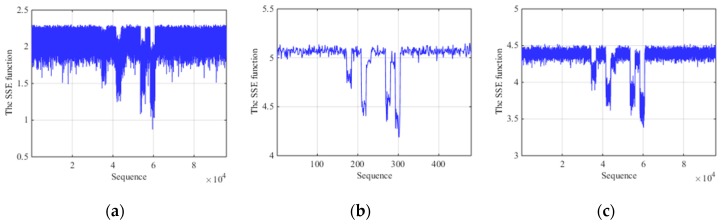
Comparison results with ISCRJ and SNR = −10 dB. (**a**) The SSE function of short fixed-scale segmentation. (**b**) The SSE function of long fixed-scale segmentation. (**c**) The SSE function of AMS.

Since the SSEF has stability to noise and jamming, we illustrate the advantages of the SSE between fixed-scale segmentation and AMS under low SNR. Figure 7a represents the SSE function of short fixed-scale segmentation with SNR = −10 dB. Figure 7b is the SSE function of long fixed-scale segmentation and Figure 7c is that of AMS with the same condition. From the figures, we can see that the SSE function of short fixed-scale segmentation are so obvious that it produces unnecessary results in terms of characteristics detection. And these fine characteristics result in the local convergence, making it difficult to distinguish between target, jamming and noise. For long fixed-scale, the sequence of the SSE function is smaller and smoother than short fixed-scale but the trend of characteristics can be seen to some extent. However, due to the relative reduction of sequence, the fine characteristics of target and jamming segments are not reflected in detail and some information will be lost for the following signal processing. So the comparison results show that the SSE function of AMS has a recognition advantage under low SNR.

### 4.2. Interference Suppression Based on SSEF

**Figure 8:**
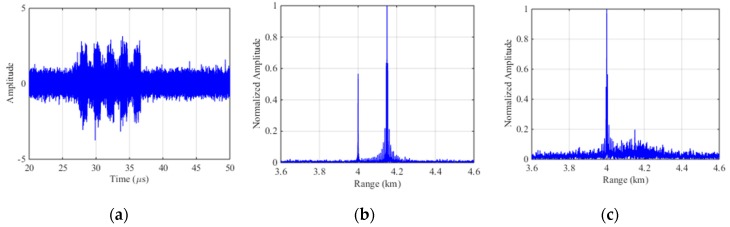
Simulation results of SSEF for ISDRJ. (**a**) The echo signal with ISDRJ; (**b**) Pulse compression of the echo signal; (**c**) Pulse compression of echo signal after SSEF.

Based on the parameters in Section 4.1, the experiments are carried out. Figure 8a represents the echo signal with ISDRJ in time domain where there are five slices in the jamming component. Figure 8b is the pulse compression result before interference suppression where the master false target occurs at 4.15 km with multiple false targets uniformly surrounded. Figure 8c is the pulse compression result after SSEF-based interference suppression without any false target which directly proves the effectiveness of the method.

**Figure 9:**
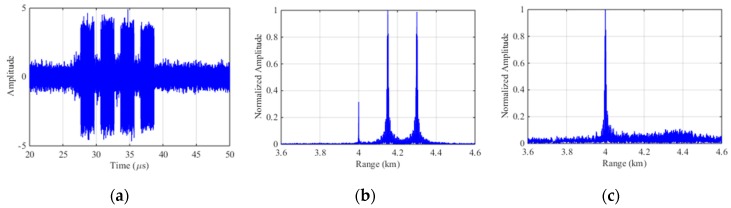
Simulation results of SSEF for ISPRJ. (**a**) The echo signal with ISPRJ; (**b**) Pulse compression of the echo signal; (**c**) Pulse compression of echo signal after SSEF.

The echo signal of ISPRJ in time domain is demonstrated in Figure 9a. Figure 9b is the pulse compression result before interference suppression where the master false target occurs at 4.15 km and 4.3 km with multiple false targets uniformly surrounded. Figure 9c is the pulse compression result after SSEF interference suppression without any false target which also proves the effectiveness of this method.

**Figure 10:**
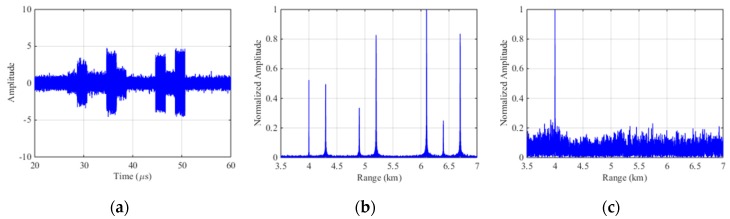
Simulation results of SSEF for ISCRJ. (**a**) The echo signal with ISCRJ; (**b**) Pulse compression of the echo signal; (**c**) Pulse compression of echo signal after SSEF.

Figure 10a is the echo signal of ISCRJ in time domain where the slices is repeating in order of sampled time and the stochastic intensive target after pulse compression is constructed by modulating the amplitude of the jamming signal. Figure 10b is the pulse compression result of the echo signal before interference suppression where three sets of false targets occurs along with the three repeater slices. The number of master false target in each set is equivalent to the number of the repeater slices and each master false target is surrounded by symmetrical false targets. Figure 10c is the pulse compression result after SSEF interference suppression without any false target which proves the effectiveness of this method.

### 4.3. Performance Comparison

As to compare the performance of different interference suppression methods, experiments are carried out between the proposed method and the STMF, the STFT, the FEDBP and the PF methods. The parameters in the experiments are the same as Section 4.1 where the probability of target detection and the PSR after pulse compression are used as evaluation criteria. According to literatures, most interference suppression methods can achieve satisfactory performance in the condition of high SNR because the characteristics of the jamming signal and the target signal are significantly different. Therefore, comparisons of the five methods in our experiment only focus on cases with SNR less than 5 dB.

**Figure 11:**
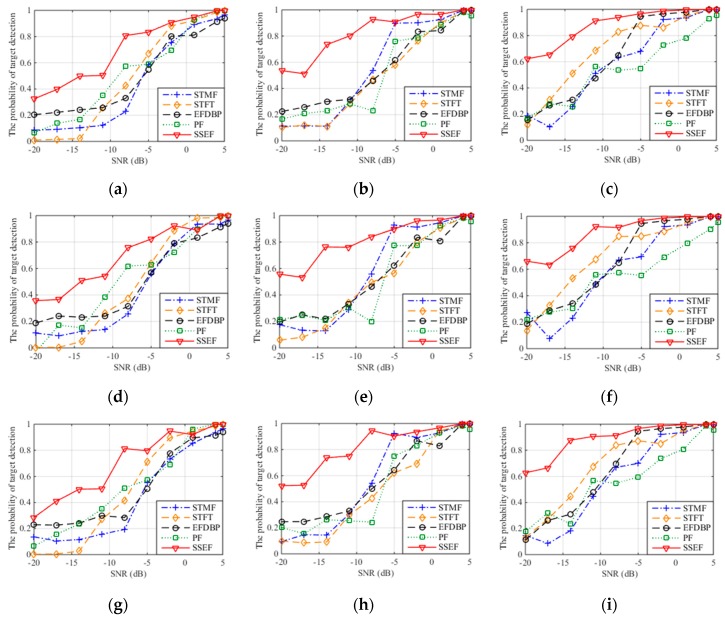
The probability of target detection with different interference suppression methods. (**a**) The probability of target detection with ISDRJ and JSR = 5 dB; (**b**) The probability of target detection with ISDRJ and JSR = 10 dB; (**c**) The probability of target detection with ISDRJ and JSR = 15 dB; (**d**) The probability of target detection with ISPRJ and JSR = 5 dB; (**e**) The probability of target detection with ISPRJ and JSR = 10 dB; (**f**) The probability of target detection with ISPRJ and JSR = 15 dB; (**g**) The probability of target detection with ISCRJ and JSR = 5 dB; (**h**) The probability of target detection with ISCRJ and JSR = 10 dB; (**i**) The probability of target detection with ISCRJ and JSR = 15 dB.

Figure 11 is the probability of target detection curve after the interference suppression with three jamming patterns and five interference suppression methods where 1000 times of Monte Carlo simulation are performed on each SNR. Table 2, Table 3 and Table 4 are the probabilities of target detection of three interference patterns with JSR = 15 dB.

Results can be first made from the aspect of jamming pattern. For the same jamming pattern, the performances of five interference suppression methods increase with the SNR, which indicates the correctness of this method and the applicability of ISRJ and its improvement pattern. When the SNR is greater than 5 dB, the target detection probability of the five methods after interference suppression is greater than 0.9. This means the proposed method is comparable to the interference suppression ability of the other methods in the condition of high SNR.

It can also be concluded from the aspect of SNR that for the same JSR, the detection probability of this method after interference suppression is greatly improved compared with other four methods when the SNR is less than 0 dB (especially between −20 dB and −10 dB). This means that the proposed method has significant advantages of interference suppression and better noise stability under low SNR conditions.

For the same SNR, when the JSR is larger than 10 dB, the detection probability after interference suppression increases with the JSR value and the difference between the jamming signal and target signal become larger as well. Meanwhile, it can be observed that in this case the detection probability and the interference suppression performance of the proposed method is higher than other four methods and there is a significant improvement of detection probability when the JSR is between 5 dB to 10 dB.

**Figure 12:**
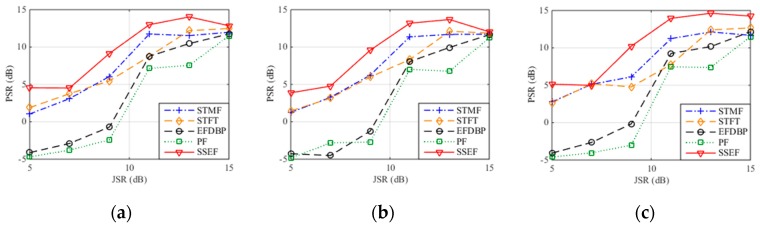
PSR after pulse compression with different interference suppression methods. (**a**) ISDRJ; (**b**) ISPRJ; (**c**) ISCRJ.

Figure 12 demonstrates the PSR of the ISDRJ, ISPRJ and ISCRJ after pulse compression with SNR = −10 dB and 1000 times of Monto Carlo simulations. It is not hard to find that the PSRs of the five methods increase with the JSR and the PSR of the proposed method is larger than other four methods which means it has the best interference suppression performance compared to the others. Table 5, Table 6 and Table 7 give the PSR values of three jamming patterns after pulse compression where the proposed method can achieve the highest PSR and the best interference suppression with −10 dB SNR and 13 dB JSR.

## 5. Conclusions

Based on the study of ISRJ principle, this paper proposed an SSEF-based ISRJ interference suppression method aiming at the problem of multiple coherence false targets in low SNRs. This method first establishes the singular spectrum model by proposed AMS, delay embedded mapping and the following SVD. Then based on SSEF derived from the Shannon entropy, this method realizes the detection of SSE function and the band-pass filtering of the echo signal received at the radar antenna. The influence of AMS and delay embedded mapping on the interference suppression performance are analyzed during the model construction. The validity and the feasibility of this method is further proved by mathematical deduction. Through the comparison with other suppression methods, the superiority of this method is finally presented from different aspects. Experiments show that in condition of low SNR, the proposed method can achieve better noise and jamming stability. Moreover, this method gives a mathematical verification and theoretical basis for the overall design of anti-jamming in ECM. Further researches will be carried out on the application of this algorithm.

## Figures and Tables

**Table 1:** Simulation parameters.

Parameter	Value
Signal-to-noise ratio SNR	0 dB
Jamming-to-signal ratio JSR	10 dB
Carrier frequency $f_{0}$	500 MHz
Pulse width T	10 μs
Frequency modulation slope K	20 MHz/μs
Target location $R_{0}$	4 km
Radar cross section of target RCS	Swerling IV
Radar sampling frequency $f_{\mathrm{sr}}$	1.2 GHz
Jammer sampling frequency $f_{s}$	1.2 GHz
Pulse width of jamming slices τ	1 μs
Pulse repetition interval of ISDRJ	2 μs
Slice number of ISPRJ $N_{\mathrm{ISPRJ}}$	2
Slice number of ISCRJ $N_{\mathrm{ISCRJ}}$	3
Repeater time of ISCRJ $M_{\mathrm{ISCRJ}}$	3
Processing time of jammer $\tau_{p}$	500 ns

**Table 2:** The probability of target detection with ISDRJ and JSR = 15 dB.

SNR (dB)	Different Interference Suppression Methods				
	STMF	STFT	EFDBP	PF	SSEF
−5	0.679	0.878	0.945	0.549	0.967
−8	0.631	0.828	0.649	0.535	0.937
−11	0.513	0.684	0.475	0.564	0.912
−14	0.258	0.513	0.311	0.259	0.792
−17	0.102	0.307	0.266	0.278	0.655

**Table 3:** The probability of target detection with ISPRJ and JSR = 15 dB.

SNR (dB)	Different Interference Suppression Methods				
	STMF	STFT	EFDBP	PF	SSEF
−5	0.694	0.848	0.958	0.553	0.964
−8	0.668	0.848	0.647	0.572	0.916
−11	0.488	0.673	0.483	0.559	0.921
−14	0.232	0.501	0.342	0.302	0.757
−17	0.076	0.327	0.288	0.275	0.633

**Table 4:** The probability of target detection with ISCRJ and JSR = 15 dB.

SNR (dB)	Different Interference Suppression Methods				
	STMF	STFT	EFDBP	PF	SSEF
−5	0.705	0.873	0.945	0.595	0.967
−8	0.673	0.837	0.695	0.547	0.912
−11	0.449	0.677	0.488	0.568	0.906
−14	0.184	0.445	0.310	0.235	0.878
−17	0.085	0.273	0.263	0.320	0.667

**Table 5:** PSR after pulse compression for ISDRJ with SNR = −10 dB.

JSR (dB)	Different Interference Suppression Methods				
	STMF	STFT	EFDBP	PF	SSEF
5	1.046	1.900	−4.123	−4.691	4.546
7	3.103	3.761	−2.894	−3.786	4.522
9	6.064	5.476	−0.706	−2.414	9.146
11	11.740	8.754	8.769	7.146	12.980
13	11.530	12.190	10.460	7.533	14.030
15	11.980	12.480	11.750	11.480	12.850

**Table 6:** PSR after pulse compression for ISPRJ with SNR = −10 dB.

JSR (dB)	Different Interference Suppression Methods				
	STMF	STFT	EFDBP	PF	SSEF
5	1.253	1.451	−4.271	−4.854	3.877
7	3.277	3.159	−4.510	−2.814	4.777
9	6.239	5.995	−1.249	−2.700	9.572
11	11.370	8.331	8.056	7.021	13.190
13	11.690	12.100	9.948	6.769	13.680
15	11.720	11.870	11.650	11.240	12.040

**Table 7:** PSR after pulse compression for ISCRJ with SNR = −10 dB.

JSR (dB)	Different Interference Suppression Methods				
	STMF	STFT	EFDBP	PF	SSEF
5	2.776	2.665	−4.075	−4.604	5.150
7	5.128	5.204	−2.645	−4.039	4.987
9	6.124	4.783	−0.165	−3.011	10.230
11	11.240	7.776	9.254	7.469	13.950
13	12.130	12.400	10.170	7.377	14.630
15	11.680	12.680	12.160	11.500	14.250

## Appendix A

According to Equations (31) and (32), as to ensure that SSEF make sense, *p_i,j_* should be non-zero which means any singular value in the singular spectrum is non-zero and the improved delay embedded matrix is full rank. Theoretically, the improved delay embedded matrix is obtained by the reconstruction mapping of the echo signal sequences received at the radar antenna which means the full rank property of the delay embedded matrix can be proved by the full rank of the echo signal sequences. Equation (18) shows that this can be further proved by matrix ***A*** and take ISDRJ as an example, the specific deduction can be summarized as follows:

$$\begin{array}{l} A=I+F_{2}\tau F_{1}P\\ =I+\left[\begin{array}{cccc} e^{j2\pi f_{1}t_{1}} & e^{j2\pi f_{2}t_{1}} & \cdots & e^{j2\pi f_{n}t_{1}}\\ e^{j2\pi f_{1}t_{2}} & e^{j2\pi f_{2}t_{2}} & \cdots & e^{j2\pi f_{n}t_{2}}\\ \vdots & \vdots & \ddots & \vdots \\ e^{j2\pi f_{1}t_{n}} & e^{j2\pi f_{2}t_{n}} & \cdots & e^{j2\pi f_{n}t_{n}} \end{array}\right]\cdot \left[\begin{array}{cccc} e^{-j2\pi f_{1}(\tau_{0}+t_{1})} & e^{-j2\pi f_{1}(\tau_{0}+t_{2})} & \cdots & e^{-j2\pi f_{1}(\tau_{0}+t_{n})}\\ e^{-j2\pi f_{2}(\tau_{0}+t_{1})} & e^{-j2\pi f_{2}(\tau_{0}+t_{2})} & \cdots & e^{-j2\pi f_{2}(\tau_{0}+t_{n})}\\ \vdots & \vdots & \ddots & \vdots \\ e^{-j2\pi f_{n}(\tau_{0}+t_{1})} & e^{-j2\pi f_{n}(\tau_{0}+t_{2})} & \cdots & e^{-j2\pi f_{n}(\tau_{0}+t_{n})} \end{array}\right]\cdot P\\ =I+\left[\begin{array}{cccc} \sum_{i=1}^{n}e^{-j2\pi f_{i}\times (\tau_{0}+0\times dt)} & \sum_{i=1}^{n}e^{-j2\pi f_{i}\times (\tau_{0}+1\times dt)} & \cdots & \sum_{i=1}^{n}e^{-j2\pi f_{i}\times (\tau_{0}+(n-1)\times dt)}\\ \sum_{i=1}^{n}e^{-j2\pi f_{i}\times (\tau_{0}-1\times dt)} & \sum_{i=1}^{n}e^{-j2\pi f_{i}\times (\tau_{0}-0\times dt)} & \cdots & \sum_{i=1}^{n}e^{-j2\pi f_{i}\times (\tau_{0}+(n-2)\times dt)}\\ \vdots & \vdots & \ddots & \vdots \\ \sum_{i=1}^{n}e^{-j2\pi f_{i}\times (\tau_{0}-(n-1)\times dt)} & \sum_{i=1}^{n}e^{-j2\pi f_{i}\times (\tau_{0}-(n-2)\times dt)} & \cdots & \sum_{i=1}^{n}e^{-j2\pi f_{i}\times (\tau_{0}-0\times dt)} \end{array}\right]\cdot P\\ =I+\sum_{i=1}^{n}e^{-j2\pi f_{i}\times \tau_{0}}\times \left[\begin{array}{cccc} \sum_{i=1}^{n}e^{-j2\pi f_{i}\times 0\times dt} & \sum_{i=1}^{n}{}^{-j2\pi f_{i}\times 1\times dt} & \cdots & \sum_{i=1}^{n}e^{-j2\pi f_{i}\times (n-1)\times dt}\\ \sum_{i=1}^{n}e^{j2\pi f_{i}\times 1\times dt} & \sum_{i=1}^{n}e^{-j2\pi f_{i}\times 0\times dt} & \cdots & \sum_{i=1}^{n}e^{-j2\pi f_{i}\times (n-2)\times dt}\\ \vdots & \vdots & \ddots & \vdots \\ \sum_{i=1}^{n}e^{-j2\pi f_{i}\times \left(n-\bar{1}\right)\times dt} & \sum_{i=1}^{n}e^{-j\bar{2}\pi f_{i}\times (n-2)\times dt} & \cdots & \sum_{i=1}^{n}e^{-j2\pi f_{i}\times 0\times dt} \end{array}\right]\cdot P\\ =I+k\times \left[\begin{array}{cccc} k_{11} & k_{12} & \cdots & k_{1n}\\ k_{21} & k_{22} & \cdots & k_{2n}\\ \vdots & \vdots & \ddots & \vdots \\ k_{n1} & k_{n2} & \cdots & k_{\mathrm{nn}} \end{array}\right]\left[\begin{array}{cccc} 1 & & & \\ & \overset{(\tau f_{s})}{\ddots } & & \\ & & 1 & \\ & & & \begin{array}{l} \begin{array}{ccc} 0 & & \end{array}\\ \begin{array}{ccc} & \overset{(\tau f_{s})}{\ddots } & \end{array}\\ \begin{array}{ccc} & & \begin{array}{cc} 0 & \end{array} \end{array}\\ \begin{array}{ccc} & & \begin{array}{cc} & \begin{array}{cc} 1 & \\ & \ddots \end{array} \end{array} \end{array} \end{array} \end{array}\right]\\ =I+k\times \left[\begin{array}{cccc} k_{11} & \overset{(\tau f_{s})}{\cdots } & k_{1,(\tau f_{s})} & \begin{array}{cccc} 0 & \overset{(\tau f_{s})}{\cdots } & 0 & \begin{array}{cc} k_{1,(2\tau f_{s}+1)} & \cdots \end{array} \end{array}\\ k_{21} & \overset{(\tau f_{s})}{\cdots } & k_{2,(\tau f_{s})} & \begin{array}{cccc} 0 & \overset{(\tau f_{s})}{\cdots } & 0 & \begin{array}{cc} k_{2,(2\tau f_{s}+1)} & \cdots \end{array} \end{array}\\ \vdots & \vdots & \vdots & \ddots \\ k_{n1} & \overset{(\tau f_{s})}{\cdots } & k_{n,(\tau f_{s})} & \begin{array}{cccc} 0 & \overset{(\tau f_{s})}{\cdots } & 0 & \begin{array}{cc} k_{n,(2\tau f_{s}+1)} & \cdots \end{array} \end{array} \end{array}\right]\\ =I+k\cdot K \end{array}$$

with:

$$K=\left[\begin{array}{cccc} k_{11} & \overset{(\tau f_{s})}{\cdots } & k_{1,(\tau f_{s})} & \begin{array}{cccc} 0 & \overset{(\tau f_{s})}{\cdots } & 0 & \begin{array}{cc} k_{1,(2\tau f_{s}+1)} & \cdots \end{array} \end{array}\\ k_{21} & \overset{(\tau f_{s})}{\cdots } & k_{2,(\tau f_{s})} & \begin{array}{cccc} 0 & \overset{(\tau f_{s})}{\cdots } & 0 & \begin{array}{cc} k_{2,(2\tau f_{s}+1)} & \cdots \end{array} \end{array}\\ \vdots & \vdots & \vdots & \ddots \\ k_{n1} & \overset{(\tau f_{s})}{\cdots } & k_{n,(\tau f_{s})} & \begin{array}{cccc} 0 & \overset{(\tau f_{s})}{\cdots } & 0 & \begin{array}{cc} k_{n,(2\tau f_{s}+1)} & \cdots \end{array} \end{array} \end{array}\right]$$

where $k=\sum_{i=1}^{n}e^{{-j2\pi f}_{i}\tau_{0}}$, $k_{\mathrm{xy}}=\sum_{i=1}^{n}e^{{-j2\pi f}_{i}(x-y)dt}$ and $dt=\frac{1}{f_{s}}$ is the sampling interval.

Assuming that λ is the eigenvalue of matrix ***K***, it can be calculated based on the eigenvalue decomposition theory that:

$$\det\left|\lambda I-K\right|={\left(-\lambda \right)}^{l\tau f_{s}}\times f(\lambda^{n-l\tau f_{s}},\ldots ,\lambda )=0$$

where *l* is the number of interrupted sampling window, ${l\tau f}_{s}$ is the total length of interrupted sampling window sequence, *n* is the total length of the signal sequence, ${n-l\tau f}_{s}$ is the total length of the repeater window sequence which represents the total length of ISRJ, ${f(\lambda }^{{n-l\tau f}_{s}},\ldots ,\lambda )$ is a function related to the eigenvalue.

The eigenvalue of matrix ***K*** can be obtained by the solution equation:

$$\lambda_{K}=\left\{\lambda_{1},\lambda_{2},\ldots ,\lambda_{n-l\tau f_{s}},\overset{l\tau f_{s}}{\overset{︷}{0,0,\ldots ,0}}\right\}$$

The eigenvalue of matrix ***A*** is:

$$\lambda_{A}=\left\{1+k\lambda_{1},1+k\lambda_{2},\ldots ,1+k\lambda_{n-l\tau f_{s}},\overset{l\tau f_{s}}{\overset{︷}{1,1,\ldots ,1}}\right\}$$

Therefore, the eigenvalues of matrix ***A*** are non-zero so as the determinant which proves the full rank property of matrix ***A*** and the validity of SSEF.
